# Dissecting gene expression in mosquito

**DOI:** 10.1186/1471-2164-12-297

**Published:** 2011-06-07

**Authors:** Rayna Stamboliyska, John Parsch

**Affiliations:** 1 Department of Biology II, University of Munich (LMU), Grosshaderner Str. 2, 82152 Planegg-Martinsried, Germany

## Abstract

Gene expression is known to vary extensively among tissues and between sexes. However, detailed descriptions of tissue- and sex-specific gene expression are available for only a few model organisms. A new study published in *BMC Genomics *presents such a data set for the mosquito, *Anopheles gambiae*, which is the vector of human malaria. In addition to providing a valuable resource for the community of mosquito researchers, the study allows comparative transcriptomic studies of dipteran insects to be extended over 250 million years of evolution, since the divergence of *A*. *gambiae *and *Drosophila melanogaster*.

## Commentary

After the genetic workhorse *Drosophila melanogaster*, the next insect to have its complete genome sequenced was the mosquito, *Anopheles gambiae *[1]. There was good reason for this attention: as the primary vector of the human malarial parasite, *A*. *gambiae *is responsible for nearly one million deaths per year [2]. Thus, there is great interest in understanding how this species interacts with both the parasite, *Plasmosium falciparum*, and its human host. A key aspect of this interaction is that only female mosquitoes take a blood meal from humans and, thus, are the only sex that serves as a vector of malaria. Males do not transmit the parasite. However, male reproduction has been a major focal point of efforts to control mosquito populations by methods such as sterile insect technique [3]. Clearly, understanding the differences between the sexes (sexual dimorphism) is critical for understanding mosquito biology. In a new study in *BMC Genomics*, Baker and colleagues [4] make a major advance in this area, using large-scale microarray analyses to quantify expression levels of over 7,000 genes in whole mosquitoes and dissected tissues of both sexes. The data have been compiled to create a detailed atlas of mosquito gene expression that is publicly available through the MozAtlas database (http://www.tissue-atlas.org).

## Sexually-dimorphic and tissue-specific gene expression in *A*. *gambiae*

Baker and colleagues used the Affymetrix microarray platform for transcriptional profiling of whole bodies, as well as eight somatic tissues (or body parts) including head, salivary gland, midgut, Malphigian tubule, testis, accessory gland, and ovary. Aside from the last three tissues, which are sex-specific, all were analyzed separately in both males and females. Overall, they observed expression of nearly 80% of the examined genes in whole bodies, with the proportion of expressed genes ranging from 51-74%, depending on the source material. The tissue showing the greatest diversity of expressed genes was the testis, which is consistent with observations from *D*. *melanogaster *[5]. In *A*. *gambiae*, ~10% of genes are expressed exclusively in testis and a further ~2% are exclusive to male accessory glands, while only ~2% of genes are expressed specifically in ovary. Outside of these reproductive tissues, the level of sexually dimorphic gene expression was still considerable, with the expression of 10-25% of genes differing between the sexes in somatic tissues.

Since only female *A*. *gambiae *feed on blood, one might expect that tissues involved in its acquisition and digestion would display sexually dimorphic gene expression. Indeed, a significantly higher number of genes expressed in the head were found to be female-biased than male-biased, including genes that encode odorant receptors. These genes are thought to play a major role in female feeding behavior, as females seeking a blood meal are attracted by human odors [6]. Another recent study that used high-throughput RNA sequencing also found differences between the sexes in the level, but not the diversity, of odorant receptor gene expression in antennae [7].

Baker and co-workers also found differences between the sexes in the functional categories of expressed genes. For example, there was an enrichment of genes involved in proteolysis and protein metabolism in the female midgut. This is consistent with the sex-limited blood feeding that provides a protein-rich diet needed for egg development. In contrast, genes showing enriched expression in the somatic tissues of males were mainly associated with carbon metabolism, which is consistent with their exclusive sugar diet.

## Chromosomal distribution of genes and comparative evolution with *D. melanogaster*

In *Drosophila*, there is a paucity of male-biased genes (both germline and somatically expressed) on the X chromosome [8]. An early microarray study of *A*. *gambiae *did not uncover such a pattern [9]. Baker and colleagues, however, did observe a strong under-representation of testis-expressed genes and a weaker (but significant) under-representation of somatic male-biased genes on the X chromosome. The paucity of X-linked male-biased genes may be the result sexually antagonistic selection and/or transcriptional inactivation of the X chromosome in the male germline [10,11].

Despite having diverged from a common ancestor 250 million years ago, one-to-one orthology could be assigned to about half of the genes present in the *A*. *gambiae *and *D*. *melanogaster *genomes. A comparison of orthologous gene expression among tissues revealed that transcription levels are similar in the midgut, carcass, and ovary. Their associated functions are also similar, supporting evolutionary conservation of gene expression networks in these organs within *Diptera*. Conversely, considerable expression differences were reported in other tissues, including testis. This is consistent with genes involved in male reproduction making a disproportionate contribution to species divergence.

## The MozAtlas database

A product of the mosquito gene expression survey that will be useful to many in the research community is the online database MozAtlas (http://www.tissue-atlas.org). Although currently not as extensive as its *Drosophila *predecessor, FlyAtlas (http://www.flyatlas.org) [5], MozAtlas allows researchers to quickly search for their gene of interest and determine its expression in various tissues of the two sexes. It also allows batch downloads and BLAST searches, which will facilitate comparative genomic analyses. MozAtlas will likely become a standard resource for the analysis of mosquito gene expression and function.
